# The Impact of Early Rehabilitation on Patients With Acute Cerebral Infarction and Chronic Kidney Disease: A Retrospective Cohort Study

**DOI:** 10.7759/cureus.98765

**Published:** 2025-12-08

**Authors:** Masahiro Nomoto, Kazuhiro Miyata, Yutaka Kohno

**Affiliations:** 1 Department of Rehabilitation, Nerimahikarigaoka Hospital, Nerima, JPN; 2 Department of Physical Therapy, Ibaraki Prefectural University of Health Sciences, Ami-machi, JPN; 3 Center for Medical Sciences, Ibaraki Prefectural University of Health Sciences, Ami-machi, JPN

**Keywords:** barthel index, chronic kidney disease, early mobilization, modified rankin scale, propensity score matching, rehabilitation

## Abstract

Background and objective

Patients with cerebral infarction (CI) who also have chronic kidney disease (CKD) are at an increased risk of adverse outcomes. However, it remains uncertain whether the presence or severity of CKD influences the effectiveness of early rehabilitation. This study aimed to investigate the impact of early rehabilitation, taking into account both the presence and severity of CKD.

Methods

This study initially included 764 patients diagnosed with CI between April 2014 and March 2021 at Nerimahikarigaoka Hospital. From this cohort, 402 patients experiencing their first CI who underwent inpatient rehabilitation were selected as the study population. Patients were categorized into two groups according to estimated glomerular filtration rate (eGFR) using the modified formula from the 2018 Evidence-based Clinical Practice Guidelines for Chronic Kidney Disease, which applies to Japanese patients: the mild group (≥45 mL/min/1.73m^2^) and the severe group (<45 mL/min/1.73m^2^). Statistical analysis involved 1:1 propensity score matching based on background factors, and standardized mean differences (SMD) were calculated. After matching, 48 patients remained in each group, due to an imbalance in baseline renal function distribution. Variables were selected based on the definition of early mobilization and factors influencing renal function decline, including age, sex, stroke type, number of stroke risk factors, and days from hospital admission to initiation of wheelchair use, standing, and gait training. Both groups were compared in terms of the primary outcomes, namely the modified Rankin Scale (mRS) and Barthel Index (BI) scores at discharge, as well as hospital stay duration.

Results

The SMDs for background factors after propensity score matching were as follows: age (0.013), time to initiation of wheelchair use (0.16), time to start of standing rehabilitation (0.071), mRS at admission (0.068), and BI at admission (<0.001). No significant differences were observed between the two groups in mRS at discharge (*p* = 0.485), BI at discharge (*p* = 0.431), or length of hospital stay (*p* = 0.226).

Conclusions

Our study revealed that early rehabilitation in CI patients consistently improves physical function, activities of daily living at discharge, and length of hospital stay, irrespective of the presence or severity of CKD. These findings highlight the clinical significance of early mobilization in CI patients with CKD.

## Introduction

Patients with cerebral infarction (CI) complicated by chronic kidney disease (CKD) have been reported to exhibit higher post-onset mortality and increased risk of poor outcomes [1-3]. CKD complications are associated with blood pressure fluctuations due to impaired cerebral autoregulation and autonomic dysfunction following CI [4,5], which can contribute to greater functional disability at discharge and prolonged hospital stays [6,7]. CI patients with CKD may experience general physical and hemodynamic instability in the immediate post-CI onset phase.

Early rehabilitation plays a crucial role in stroke management [8,9] and is typically defined as initiating out-of-bed activities, sitting, standing, walking, and other exercises targeting activities of daily living (ADLs) within 48 hours of onset [10]. Post-stroke rehabilitation services help decrease patient mortality and physical care needs [11]. Early mobilization can effectively reduce hospital stay durations and bed-related complications and also improve physical function and ADLs, indicating that it can be recommended for patients with acute CI [8,9,11,12]. Intensive rehabilitation combined with appropriate risk management can further improve functional outcomes in patients with complications. Therefore, it is hypothesized that patients experiencing their first CI who also have CKD may present with a worse general condition than those without CKD, but early mobilization could help accelerate improvements in function and ADLs.

A previous study reported that greater CKD severity is associated with higher rates of death and dependence (modified Rankin Scale (mRS) score ≥3) within three months after CI onset [13]. Furthermore, Additionally, rehabilitated patients with more severe CKD demonstrate lower ADL function six months post-CI [14]. In particular, an estimated glomerular filtration rate (eGFR) ≤45 mL/min/1.73m^2^ or lower has been shown to significantly impact early mortality, emphasizing the importance of early intervention and management in patients with advanced CKD [15].

While early rehabilitation is known to improve outcomes in stroke patients, and CKD is linked to poorer post-stroke recovery, there is limited evidence directly comparing the effects of early rehabilitation across varying CKD severity in patients with CI. Because early mobilization can effectively improve physical function and ADLs, we believe that the results of this study may help optimize patients’ functional status and ADL performance during the acute post-CI period. Additionally, early mobilization in these patients may shorten hospital stays and prevent functional declines, as determined by ADL performance postdischarge. This study, therefore, aimed to evaluate whether early mobilization influences mRS scores, Barthel Index (BI) scores, and length of hospital stay in patients with acute CI across varying CKD severity levels.

## Materials and methods

Study design and patients

This retrospective cohort study was conducted after receiving approval from the Research Ethics Committee of Nerimahikarigaoka Hospital (approval no. 21120901). Due to the study’s retrospective design, the ethics committee waived the requirement for informed consent from patients.

The patients included in this study comprised 764 individuals diagnosed with CI from April 2014 to March 2021 at Nerimahikarigaoka Hospital. The inclusion criteria included patients aged 20 years or older who received physical rehabilitation services during hospitalization for their first CI. The exclusion criteria were as follows: a history of previous stroke, recurrent stroke during hospitalization, lack of gait rehabilitation, in-hospital death, ongoing dialysis, thrombolytic therapy, endovascular procedures or surgery during hospitalization, and complications from infections or symptom exacerbation. All patients received standard rehabilitation programs provided by licensed therapists during hospitalization. Early mobilization was defined, based on previous studies, as initiation of wheelchair use within 48 hours [10].

Data extraction and outcomes

Data for this study were obtained from the medical records of the included patients, which were accessed between December 25, 2021, and March 22, 2022. After the required information was collected, no further access to the records was performed. Variables were selected based on the definition of early mobilization (e.g., sitting, standing, walking), factors influencing functional outcomes after CI, and factors affecting renal function decline [10,16-18]. Collected data included age, sex, stroke type, eGFR, number of stroke risk factors, and the number of days from hospital admission to initiation of wheelchair use, standing, and gait rehabilitation. Renal function was assessed using eGFR values automatically reported in the hospital’s laboratory test results at admission.

We used the mRS, BI, and hospital stay duration as our primary outcomes, in line with previous research [12,16]. The mRS is the most widely used functional assessment tool in the acute phase of stroke and has demonstrated good reliability [19,20]. The mRS is graded as follows: 0 = no symptoms; 1 = no significant disability despite symptoms, able to perform all usual activities; 2 = slight disability, unable to carry out all previous activities but able to manage personal affairs without assistance; 3 = moderate disability, requiring some help but able to walk without assistance; 4 = moderately severe disability, unable to walk or attend to bodily needs without help; 5 = severe disability, bedridden, incontinent, and requiring constant nursing care; and 6 = death.

The BI is the standard ADL assessment tool, and has demonstrated good reliability [19,21]. The BI measures ADL performance and assesses specific mobility and self-care activities: feeding, personal toileting, bathing, dressing and undressing, moving on and off a toilet, controlling the bladder, controlling bowel movements, transferring from a wheelchair to bed and back, walking on a level surface, and ascending and descending stairs. The BI score range comprises 0-100 [22]. mRS and BI were assessed by trained rehabilitation therapists (physical, occupational, and speech-language therapists) at admission and at the final rehabilitation session on the day before discharge. Data were extracted by a single investigator using a predefined set of study variables and a self-designed data collection sheet. All required variables were available in the medical records, and no missing data were present for the included patients.

Statistical analysis

For statistical purposes, we categorized the patients into two groups: the mild group, with normal to mildly impaired renal function (eGFR ≥45 mL/min/1.73 m²), and the severe group, with moderate to severe renal impairment (eGFR <45 mL/min/1.73 m²) [23,24]. Standard eGFR calculation methods, including the Modification of Diet in Renal Disease and CKD Epidemiology Collaboration formulas, are widely used for global assessments [23,24]; however, these formulas are less accurate when eGFR is below 60 mL/min/1.73 m², highlighting the need for a Japan-specific formula [25,26]. Accordingly, this study utilized the modified eGFR formula from the 2018 Evidence-based Clinical Practice Guideline for CKD, which is specifically validated for Japanese patients.

We calculated propensity scores after adjusting for the background factors of age, days between hospital admission and the initiation of wheelchair and standing rehabilitation, and mRS and BI scores at admission. These adjustments were guided by the definitions of early mobilization and factors impacting prognosis post-CI onset [10,18]. We excluded the number of days between hospital admission and gait rehabilitation initiation, as this measure is often influenced by physicians’ orders or patients’ overall condition. Logistic regression was used to calculate the propensity score, with background factors as the independent variables and eGFR severity as the dependent variable. A receiver operating characteristic (ROC) curve was drawn based on the propensity score, and the c-statistic was calculated.

Finally, propensity score matching was conducted using one-to-one nearest-neighbor matching with a caliper of 0.2 and without replacement. Standardized mean difference (SMD) was calculated for matched data, with SMDs <0.1 indicating a balanced distribution. Postmatching data, including mRS and BI at discharge, hospital stay duration, and recovery rates, were compared using the paired t-test. All statistical analyses were performed using R version 4.3.2 (Foundation for Statistical Computing, Vienna, Austria), with a significance threshold of p < 0.05. The study utilized the pROC, Matching, and tableone packages.

## Results

**Figure 1:**
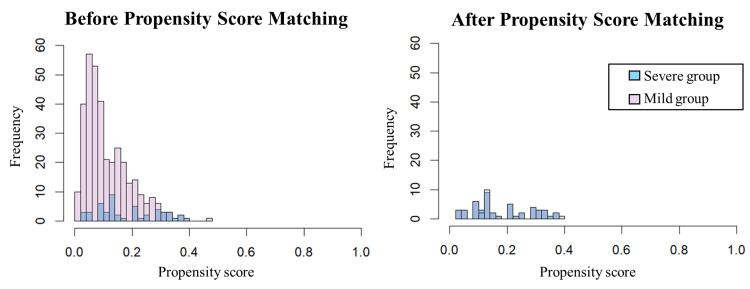
Distributions before and after propensity score matching Mild group: eGFR ≥45 mL/min/1.73m^2^ [23,24]; severe group: eGFR <45 mL/min/1.73m^2^ [23,24] eGFR: estimated glomerular filtration rate

We identified 402 patients from an initial pool of 764. Before propensity score matching, there were 353 in the mild and 49 in the severe groups (Table 1). Table 2 shows the SMD values for background factors: age (SMD = 0.74), days between hospital admission and wheelchair ride initiation (SMD = 0.36), days between hospital admission and standing rehabilitation initiation (SMD = 0.39), mRS at admission (SMD = 0.43), and BI at admission (SMD = 0.55). The c-statistic for the propensity score’s ROC curve was 0.71 (95% confidence interval (CI) = 0.63-0.79), indicating strong discriminatory power. The distribution of matches pre- and post-propensity score application is depicted in Figure 1.

**Table 1:** Clinical characteristics of the patients ESUS: embolic stroke of undetermined sources; eGFR: estimated glomerular filtration rate [23,24]; mRS: modified Rankin scale [19,20]; BI: Barthel Index [22]; SD: standard deviation

Characteristic	Before propensity score matching (n = 402)	After propensity score matching (n = 96)
Age, years, mean ± SD	72.8 ± 12.6	80.0 ± 10.8
Sex, male, n (%)	256 (63.7)	40 (58.3)
Type of stroke, n (%)		
Atheroma	147 (36.6)	44 (45.8)
Cardiogenic	40 (10.0)	15 (15.6)
Lacunar	151 (37.6)	24 (25.0)
ESUS	64 (15.9)	13 (13.5)
The number of stroke risk factors, mean ± SD	1.9 ± 1.1	2.0 ± 1.1
eGFR at admission, mL/min/1.73m^2, ^mean ± SD	64.8 ± 18.8	51.6 ± 22.7
Wheelchair ride initiation, days, mean ± SD	1.4 ± 1.1	1.8 ± 1.4
Standing rehabilitation initiation, days, mean ± SD	2.0 ± 1.2	2.4 ± 1.5
Gait rehabilitation initiation, days, mean ± SD	2.5 ± 2.9	3.5 ± 4.1
Hospital length of stay, days, mean ± SD	17.4 ± 12.5	21.1 ± 18.7
mRS at admission, median (range)	4 (4–4)	4 (4–4.25)
BI at admission, points, median (range)	55 (25–75)	30 (10–60)

**Table 2:** Clinical characteristics after propensity score matching by severity ^*^Wilcoxon rank-sum test. ^†^Pearson’s chi-squared test. ^‡^Fisher’s exact test. ^§^Paired t-test Mild group: normal to mild group [23,24]; severe group: moderate to severe group [23,24]; ESUS: embolic stroke of undetermined sources; eGFR: estimated glomerular filtration rate [23,24]; mRS: modified Rankin scale [19,20]; BI: Barthel Index [22]; SMD: standardized mean difference; SD: standard deviation

	Before propensity score matching					After propensity score matching				
	Mild group (n = 353)	Severe group (n = 49)	Test statistic	p	SMD	Mild group (n = 48)	Severe group (n = 48)	Test statistic	p	SMD
Age, years, mean ± SD	71.8 ± 12.6	80.2 ± 10.2	W = 5254^*^	<0.001	0.735	80.0 ± 11.4	80.0 ± 10.3	t = -0.066^§^	0.948	0.013
Sex, male, n (%)	225 (63.7)	31 (63.3)	χ^2^ < 0.001^†^	1.000	0.010	26 (54.2)	30 (62.5)	χ^2^ = 0.386^†^	0.535	0.170
Type of stroke, n (%)										
Atheroma	122 (34.6)	25 (51.0)	χ^2^ = 4.341^†^	0.037	0.337	19 (39.6)	25 (52.1)	χ^2^ = 1.049^†^	0.306	0.253
Cardiogenic	33 (9.3)	7 (14.3)	Fisher^‡^	0.408	0.153	9 (18.8)	6 (12.5)	χ^2^ = 0.316^†^	0.574	0.173
Lacunar	143 (40.5)	8 (16.3)	χ^2^ = 9.723^†^	0.002	0.557	16 (33.3)	8 (16.7)	χ^2^ = 2.722^†^	0.099	0.392
ESUS	55 (15.6)	9 (18.4)	χ^2^ = 0.085^†^	0.771	0.074	4 (8.3)	9 (18.8)	χ^2^ = 1.424^†^	0.233	0.308
The number of stroke risk factors, mean ± SD	1.9 ± 1.1	2.1 ± 1.1	W = 8245.5^*^	0.420	0.123	2.0 ± 1.1	2.0 ± 1.1	t = -0.385^§^	0.701	0.079
eGFR at admission, mL/min/1.73m^2, ^mean ± SD	69.2 ± 15.2	33.0 ± 9.2	W = 17297^*^	<0.001	2.868	70.1 ± 15.8	33.1 ± 9.4	t = 14.0^§^	<0.001	2.856
Wheelchair ride initiation, days, mean ± SD	1.4 ± 1.1	1.8 ± 1.6	W = 7154^*^	0.006	0.358	1.9 ± 1.5	1.7 ± 1.3	t = 0.792^§^	0.430	0.162
Standing rehabilitation initiation, days, mean ± SD	1.9 ± 1.1	2.5 ± 1.6	W = 6956^*^	0.003	0.392	2.5 ± 1.5	2.4 ± 1.5	t = 0.346^§^	0.730	0.071
Gait rehabilitation initiation, days, mean ± SD	2.3 ± 1.9	4.1 ± 6.5	W = 7016^*^	<0.001	0.385	3.5 ± 3.1	3.5 ± 5.0	t = -0.073^§^	0.942	0.015
Length of hospital days, days, mean ± SD	16.5 ± 9.8	23.7 ± 23.6	W = 6829^*^	<0.001	0.402	18.8 ± 11.5	23.5 ± 23.8	t = -1.220^§^	0.226	0.249
mRS at admission, median (range)	4 (0–5)	4 (2–5)	W = 6730^*^	0.008	0.430	4 (2–5)	4 (2–5)	t = 0.333^§^	0.740	0.068
BI at admission, points, median (range)	40 (0–95)	30 (0–85)	W = 11260^*^	<0.001	0.547	30 (0–85)	32.5 (0–85)	t = 0^§^	1.000	<0.001

**Figure 2:**
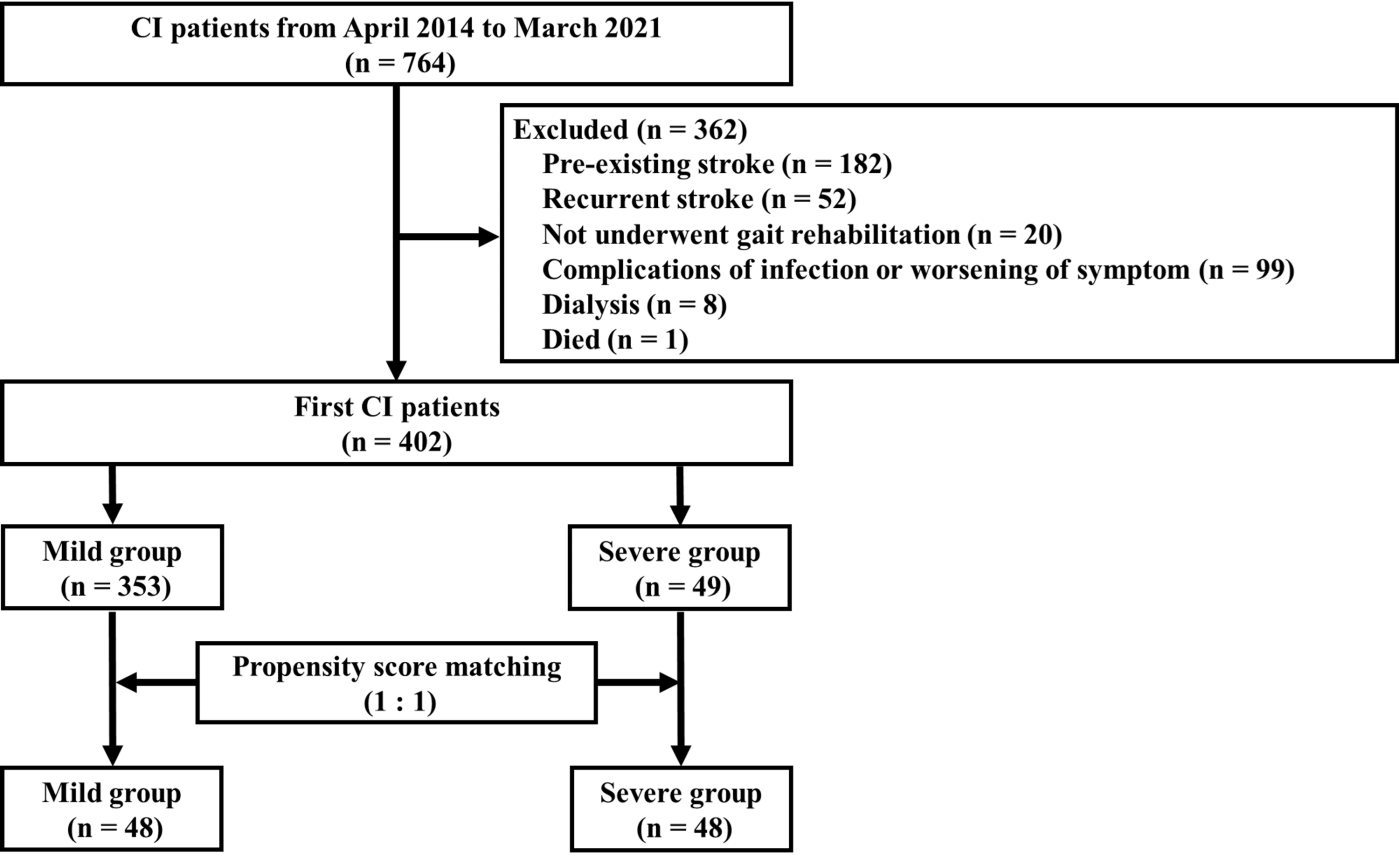
Flow chart depicting patient selection and propensity score matching Mild group: eGFR ≥45 mL/min/1.73m^2^ [23,24]; severe group: eGFR <45 mL/min/1.73m^2^ [23,24] CI: cerebral infarction; eGFR: estimated glomerular filtration rate

After propensity score matching, the study cohort comprised 48 in the mild group and 48 in the severe group. We excluded 305 patients classified as “mild” and one patient as “severe” (Table 2). The patient selection flow is shown in Figure 2, and Table 2 outlines the SMD values for background factors postmatching: age (SMD = 0.013), days between hospital admission and wheelchair ride initiation (SMD = 0.16), days between hospital admission and standing rehabilitation initiation (SMD = 0.071), mRS at admission (SMD = 0.068), and BI at admission (SMD < 0.001).

We found no significant between-group differences for mRS and BI at discharge or hospital stay duration. Furthermore, the mRS and BI recovery rates did not significantly differ between the groups (Table 3).

**Table 3:** Comparison of mRS and BI scores at discharge and hospital length of stay for patients in the mild and severe (based on baseline eGFR) groups ^*^Wilcoxon rank-sum test. ^†^Paired t-test Background factors included age, wheelchair rides-start days, standing rehabilitation-start days, mRS at admission, and BI at admission Mild group: normal to mild group [23,24]; severe group: moderate to severe group [23,24]; mRS: modified Rankin scale [19,20]; BI: Barthel Index [22]; SD: standard deviation

Variables	Before propensity score matching				After propensity score matching			
	Mild group (n = 353)	Severe group (n = 49)	Test statistic	p	Mild group (n = 48)	Severe group (n = 48)	Test statistic	p
mRS at discharge, median (range)	2 (0–5)	3 (0–5)	W = 6220.5^*^	0.001	2.5 (0–4)	3 (0–5)	t = -0.700^†^	0.485
The degree of mRS recovery, median (range)	2 (0–5)	1 (0–5)	W = 10383^*^	0.044	1 (0–4)	1 (0–5)	t = 0.906^†^	0.367
BI at discharge, points, median (range)	95 (5–100)	85 (0–100)	W = 11347^*^	<0.001	90 (5–100)	85 (5–100)	t = 0.791^†^	0.431
The degree of BI recovery, points, median (range)	30 (0–100)	30 (0–100)	W = 8844^*^	0.842	30 (5–100)	32.5 (0–100)	t = 0.947^†^	0.346
Length of hospital days, days, mean ± SD	16.5 ± 9.8	23.7 ± 23.6	W = 6829^*^	<0.001	18.8 ± 11.5	23.5 ± 23.8	t = -1.220^†^	0.226

## Discussion

We aimed to determine whether early mobilization differentially affected physical function, ADL outcomes, or hospitalization duration in CI patients based on their CKD status. The results of propensity score matching showed no significant difference in mRS and BI at discharge and hospital stay between the mild and severe groups. Early mobilization for patients with acute CI was indicated to improve functional condition, ADL at discharge, and hospital stay, regardless of CKD status. As in previous studies, the pre-propensity score matching results in this study showed that the severe group had significantly worse mRS and BI at discharge and longer hospital stays [6,7,27]. However, there were no significant between-group differences after adjusting for background factors (age, days between hospital admission and the initiation of wheelchair and standing rehabilitation, and mRS and BI scores at admission).

Early rehabilitation is an essential part of stroke care [8,9], and early mobilization (within 24-48 hours) has been shown to influence mortality, neurological deterioration, and fall risk [16]. A systematic review of early mobilization in stroke patients reported that it was not associated with increased bed-related complications and was effective in reducing hospital stay and improving ADLs [12]. In this study, both groups underwent wheelchair mobilization within 48 hours of onset (1.9 ± 1.5 vs. 1.7 ± 1.3 days). These findings suggest that early mobilization for CI patients with CKD may enhance mRS and BI scores at discharge without prolonging hospitalization.

The number of patients before propensity score matching was 353 in the mild group and 49 in the severe group; however, after propensity score matching, the number of patients in the mild group decreased significantly to 48. The mild group was characterized by younger age, had a higher proportion of lacunar infarctions, initiated wheelchair, standing, and gait rehabilitation earlier, experienced shorter hospital stays, and had higher ADL scores at admission. Lacunar infarctions are indicative of small vessel disease, and symptoms may often be minimal or absent. Because age and ADL performance influence physical function recovery [18,28], the mild group likely included more patients with mild or milder illnesses. Additionally, these patients might enjoy a better post-onset course, making it difficult to accurately compare mRS and BI at discharge and hospital stay before propensity score matching is performed. This indicates that the poorer outcomes observed in the severe group before matching were largely due to baseline differences rather than the effect of early mobilization itself. Nevertheless, propensity score matching may attenuate clinically relevant differences, so the results should be interpreted cautiously.

A key finding of this study is that early mobilization enhances functional status and ADL performance in patients with acute CI and coexisting CKD, similar to its effects in patients without CKD or with only mild CKD, without prolonging the length of hospital stay. Although complications of CKD affect functional condition and ADL decline after CI onset [5-7,14,29], the results suggest that early mobilization with appropriate risk management can improve physical functional status and ADL performance, regardless of CKD severity. Therefore, in the early mobilization of CI patients with CKD, it is important to carefully assess blood pressure variability during mobilization and rehabilitation, and to determine the appropriate mobilization and rehabilitation load settings in consultation with the attending physician.

Our study has several major limitations. Firstly, this study only included patients with a first CI. The impact of early mobilization on patients with a history of or recurrent CI, which was set as an exclusion criterion, has not been fully investigated. Secondly, the number of patients in the severe group was small, and the sample size in the mild group decreased substantially after propensity score matching. Moreover, certain baseline imbalances remained, as the SMD for the number of days until wheelchair rides did not fall below 0.1. These issues indicate that our between-group comparisons may be insufficiently robust, and the findings should be interpreted with caution to avoid overgeneralization. In addition, as this was a single-center study, the external validity of our findings is limited. Thirdly, the retrospective design restricts causal interpretation, and potential residual confounders may still influence the results despite matching. Finally, the effects of dialysis were not examined in detail because our study population included patients with normal renal function and patients with CKD who did not undergo dialysis. Patients undergoing dialysis are more prone to functional declines than their nondialysis counterparts [30]. Moreover, more severe renal impairment may mechanistically contribute to slower recovery [13-15].

Thus, while our findings suggest potential benefits of early mobilization regardless of CKD status, these methodological constraints must be considered when interpreting the results. Future large-scale, prospective studies are warranted to confirm and expand upon our observations, including in patients with more severe baseline conditions.

## Conclusions

Regardless of the presence or extent of CKD, no significant differences in physical functional status or ADL at discharge were observed in patients with acute CI who underwent early rehabilitation. Despite the generally poor prognosis of patients with CI and CKD, our findings suggest that early rehabilitation may improve outcomes. Future studies should examine whether early mobilization can enhance mRS and BI in dialysis patients to a similar degree as in patients who are not on dialysis.
